# Feasibility and preliminary diagnostic results of pixel-wise quantification of regadenoson first pass cardiac magnetic resonance perfusion imaging

**DOI:** 10.1186/1532-429X-16-S1-P214

**Published:** 2014-01-16

**Authors:** Allison D Ta, Li-Yueh Hsu, Christopher A Miller, Anders M Greve, Hannah Conn, Susanne Winkler, Peter Kellman, Kim-Lien Nguyen, Sujata M Shanbhag, Marcus Y Chen, W Patricia Bandettini, Andrew E Arai

**Affiliations:** 1 NHLBI, Bethesda, Maryland, USA; 2 Duke University School of Medicine, Durham, Maryland, USA

## Background

In this study we evaluate the diagnostic accuracy of myocardial perfusion pixel maps for detecting coronary artery disease (CAD). We also compare the performance of perfusion pixel maps with and without myocardial segmentation.

## Methods

Forty-seven patients had 1.5T steady state free precession, regadenoson stress perfusion CMR and either a ≤ 30% coronary narrowing by CT angiography (CTA) or a ≥70% stenosis by invasive coronary angiography (ICA). CMR perfusion pixel maps were generated by a model-constrained deconvolution with and without the myocardial segmentation. The two independent observers were blinded to clinical information.

## Results

**Figure 1:**
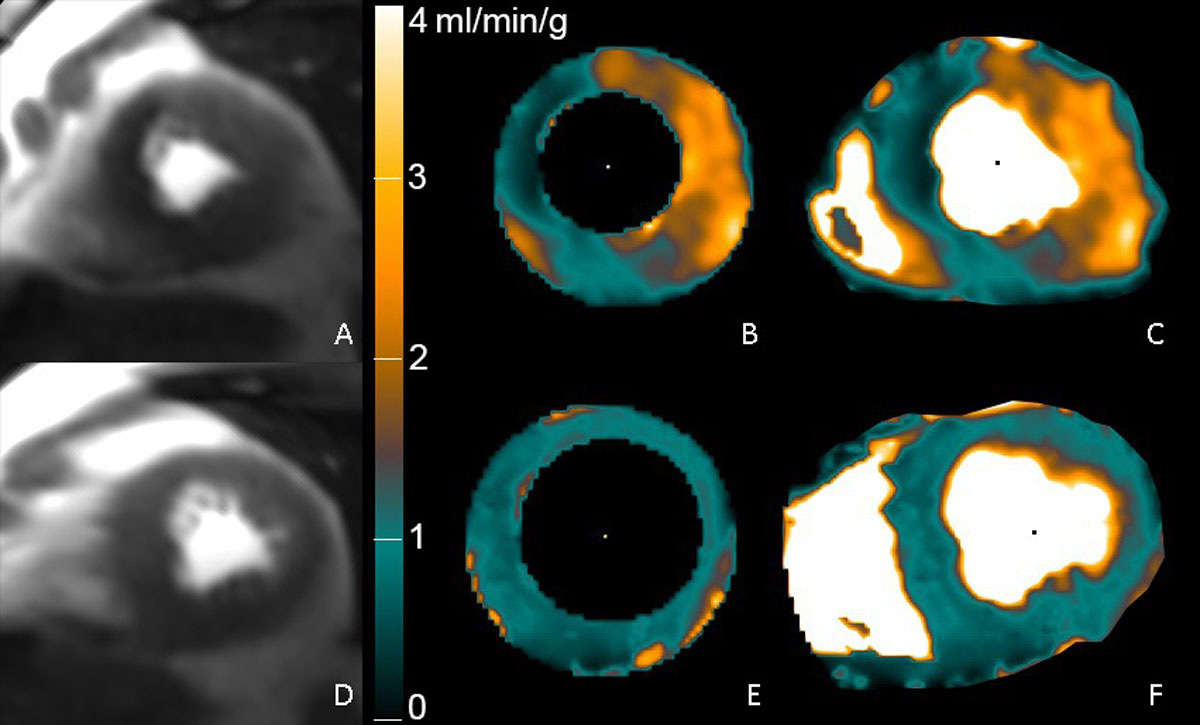
**Perfusion Pixel-Maps in a patient with 85% LAD and RCA stenoses**. A) stress perfusion image, B) pixel map generated using Epi/Endo ROIs, C) pixel map generated without ROI's. D), E) and F) are the rest images and perfusion maps.

Significant coronary stenoses were present in 22 of 47 patients. The diagnostic accuracy of pixel-wise quantification of myocardial perfusion is summarized in Table 1. All of the pixel maps were of diagnostic quality (Figure 1). Interobserver agreement was excellent for ROI guided pixel maps (kappa 0.87). Agreement between readers for perfusion pixel-maps made without ROIs was kappa = 0.59. One reader tended towards higher sensitivity while the other reader favored specificity.

**Table 1:** Diagnostic Accuracy of Pixel-wise Quantification of Regadenoson Perfusion

		Sensitivity	Specificity	Accuracy	PPV	NPV
No ROIs	Obs 1	0.82	0.88	0.85	0.86	0.85

	Obs 2	0.95	0.6	0.77	0.68	0.94

Epi/Endo ROIs	Obs 1	0.77	0.96	0.87	0.94	0.83

	Obs 2	0.86	0.92	0.89	0.9	0.88

## Conclusions

Fully quantitative stress perfusion analysis at a pixel level has significant potential in clinical settings. As evidence of feasibility, diagnostic quality perfusion pixel maps could be generated without the spatial constraints of the epicardial and endocardial ROIs.

## Funding

ADT is supported by the Sarnoff Cardiovascular Research Foundation. CM is supported by the National Institutes of Health Research, UK. ADT, LH, HC, SW, KN, PK, SS, MC, PB, and AA are supported by the National Institutes of Health, USA.

